# The changing landscape of head and neck cancer radiotherapy patients: is high‐risk, prolonged feeding tube use indicative of on‐treatment weight loss?

**DOI:** 10.1002/jmrs.349

**Published:** 2019-08-06

**Authors:** Nigel J. Anderson, James E. Jackson, Morikatsu Wada, Michal Schneider, Michael Poulsen, Maureen Rolfo, Maziar Fahandej, Hui Gan, Vincent Khoo

**Affiliations:** ^1^ Department of Radiation Oncology, Olivia Newton John Cancer Wellness & Research Centre Austin Health Heidelberg Victoria Australia; ^2^ Department of Radiation Oncology and Cancer Imaging, Peter MacCallum Cancer Centre Melbourne Victoria Australia; ^3^ Department of Medical Imaging and Radiation Sciences Monash University Monash Victoria Australia; ^4^ School of Medicine Griffith University Gold Coast Queensland Australia; ^5^ Faculty of Health Sciences and Medicine Bond University Gold Coast Queensland Australia; ^6^ Radiation Oncology Centres Gold Coast University Hospital Gold Coast Queensland Australia; ^7^ Faculty of Medicine The University of Queensland Herston Queensland Australia; ^8^ Department of Palliative Care St Vincent’s Hospital Fitzroy Victoria Australia; ^9^ Department of Medical Oncology Austin Health and Olivia Newton‐John Cancer Research Institute Melbourne Victoria Australia; ^10^ School of Cancer Medicine La Trobe University School of Cancer Medicine Melbourne Victoria Australia; ^11^ Department of Medicine University of Melbourne Melbourne Victoria Australia; ^12^ Department of Clinical Oncology Royal Marsden NHS Foundation Trust and Institute of Cancer Research Chelsea, London UK

**Keywords:** feeding tube, head and neck cancer, intensity modulated radiotherapy, toxicity, weight loss

## Abstract

**Introduction:**

Precision radiotherapy relies heavily on optimal weight management. Our group previously developed a risk stratification model for patients at risk of prolonged feeding tube (FT) intervention. The study objective was to assess on‐treatment weight loss according to stratified risk of prolonged FT use.

**Methods:**

One hundred and one (*n* = 101) definitive head and neck radiotherapy patients were included in this study. Patients were stratified into high risk (HRi: T‐classification ≥ 3 with level 2 Nodal disease), high‐intermediate risk (HIRi: T‐classification ≥ 3 without level 2 Nodes) and low‐intermediate risk (LIRi: T‐classification < 3 with level 2 Nodes) of prolonged FT use. Demographic variables and on‐treatment weight loss were evaluated according to risk status.

**Results:**

Oropharyngeal carcinoma (OPC) was present in a larger proportion in the LIRi cohort (HRi: 71%, HIRi: 52%, LIRi: 81%, *P* = 0.008). LIRi patients were more likely to have human papilloma virus (HPV)‐associated disease (88%, *P* = 0.001). Never/minimal smoking (*P* = 0.003), good performance status (*P* < 0.001), healthy BMI (*P* = 0.050) and no pre‐existing dysphagia (*P* < 0.001) were predominant within the LIRi prognostic group. LIRi patients lost significantly more weight in total (HRi = 4.8% vs. LIRi = 8.2%, *P* = 0.002; HIRi = 5.2% vs. LIRi = 8.2%, *P* = 0.006) and when using a FT (HRi = 4.6% vs. LIRi = 8.8%, *P* < 0.001; HIRi = 5.3% vs. LIRi = 8.8%, *P* = 0.002).

**Conclusions:**

Patients identified as low‐intermediate risk of prolonged, ≥25% FT use report significantly increased weight loss compared with patients at higher risk of FT use. This cohort is typical of the increasing number of patients presenting with HPV‐associated OPC. Results of this study suggest we should closely observe such patients throughout treatment, to ensure optimal weight maintenance, facilitating precision radiotherapy.

## Graphical abstract

Weight loss in head and neck cancer (HNC) patients is critical in delivering precision radiation therapy. Feeding tubes are a commonly used intervention to manage nutrition during radiotherapy. The results of this study show that HPV‐associated HNC patients, whilst not typically at highest risk of prolonged feeding tube use during radiotherapy, present with increased weight loss throughout their radiotherapy.

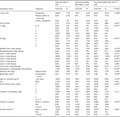

## Introduction

Radiotherapy (RT) for head and neck cancer is associated with the debilitating toxicities of malnutrition and weight loss.^1^, ^2^, ^3^ It has been long established that dysphagia and subsequent weight loss during treatment can have a detrimental effect on survival outcomes.^4^ Weight maintenance is critical to optimal treatment tolerance and paramount to the delivery of precision radiotherapy, as changes in patient contour will impact on the design and delivery of radiotherapy. Enteral feeding via a feeding tube (FT) is a common method of minimising weight loss by providing patient nutrition during and immediately following RT in as many as 80% of head and neck cancer patients.^5^, ^6^, ^7^ Despite the demonstrated benefits of FT for nutritional support, conflicting evidence remains as to the most effective strategy for optimal weight management.^8^ The previous work from our group proposed a risk stratification model for patients at risk of requiring prolonged FT use for ≥25% of nutritional requirement, to ensure insertion of prophylactic FT is reserved for those patients likely to derive the most benefit.^9^ Multivariate regression was undertaken on clinical variables previously recognised in the literature. Additional variables that were deemed to be of potential importance (i.e. specific levels of macroscopic nodal involvement) or where little to no published data were available as to their role in feeding tube risk stratification, warranting further investigation (i.e. human papilloma virus or HPV status), were also included for analysis.^10^, ^11^, ^12^, ^13^, ^14^ T‐classification and level 2 lymphadenopathy were found to be highly predictive of feeding tube use. Four levels of prolonged feeding tube use risk were derived from these two prognostic variables:

1. High risk (HRi) – T‐classification ≥ 3 and level 2 Lymphadenopathy
2. High‐intermediate risk (HIRi) – T‐classification ≥3 and No level 2 Lymphadenopathy
3. Low‐Intermediate Risk (LIRi) – T‐classification <3 and level 2 Lymphadenopathy
4. Low risk (LRi) – T‐classification <3 and No level 2 Lymphadenopathy

The main objective of this study was to assess acute weight loss (i.e. weight lost during the radiotherapy treatment course) among the high, high‐intermediate and low‐intermediate risk cohorts, to better understand whether the known risk of prolonged FT use for ≥25% of nutritional requirement is indicative of on‐treatment weight loss outcomes, and consequently, optimal FT utilisation is occurring to ensure weight maintenance during radiotherapy. Subsequently, identification of high frequency clinical variables (beyond T‐classification and level 2 lymphadenopathy) in the increased weight loss cohort will enable a greater understanding of the feeding tube use/weight loss relationship and its impact on the management of the head and neck radiotherapy patient population.

## Methods and Materials

### Patients

Following Institutional Ethics Committee approval, one hundred and one patients, treated between January 2007 and December 2013 who were previously incorporated into the already published FT risk stratification model, were included for further analysis in this retrospective study. As this study was a retrospective review of data captured as part of routine patient care, which is de‐identified, a waiver of consent was approved by the institutional ethics committee. LIRi, HIRi and HRi patients are defined as patients who have a median FT use of ≥25% of their nutritional requirement for 75, 108 and 170 days, respectively.^9^ Patients at low risk (LRi) of FT use (i.e. median feeding tube use of ≥25% of nutritional requirement of 7 days) were excluded from the analysis due to negligible likelihood of FT insertion as derived from our previous work. Patients were included in the weight loss analysis based on their risk‐stratified status alone, regardless of FT insertion or not (e.g. a HRi patient may have declined a FT insertion, yet still be included in the analysis as a ‘high‐risk’ patient due to having a T‐classification ≥3 and level 2 lymphadenopathy).

As per our previous study, to be eligible for inclusion in the database, patients were required to receive primary and definitive intensity modulated radiotherapy (IMRT) (with or without concurrent systemic treatment) for mucosal cancers of the head and neck.^9^ Patients with stage II–IVB disease were included. Patients were excluded if they underwent therapeutic surgery to the primary site or neck dissection prior to commencing RT. Patients were required to have been offered a prophylactic FT prior to the treatment (as per departmental policy), have a tumour of supraglottic, oral cavity or pharyngeal origin and planned to receive ≥64Gy with bilateral nodal irradiation, with or without current chemotherapy. ‘FT only’ patients are defined as those who had a FT inserted and utilised it for >25% of nutritional needs (for at least 48 h), as opposed to those who did not have a FT inserted (declined) and/or patients who had a FT inserted and did not use it (i.e. failed to utilise their FT for more than 25% of their nutritional needs for at least 48 h). Patients with unknown primary and glottic laryngeal cancers were excluded from the risk stratification model. All included patients had to be followed up by a dietician for a minimum of 8 weeks post‐radiotherapy completion.

### RT planning and treatment

Uniform delineation of all radiotherapy target volumes was performed by a (one) radiation oncologist on a radiotherapy planning contrast‐enhanced computed tomography (CECT) scan. The PET/CT and MRI (if available) were co‐registered with the planning CECT on the treatment planning system. A comprehensive narrative detailing target delineation, radiation dose, radiotherapy planning and treatment methodology is described in Anderson et al.^9^

### Nutritional assessment and follow‐up

All patients had a complete pre‐therapy consultation with a dietician followed by weekly nutritional reviews while on therapy. Following therapy, dietetic review, by phone or in person, was conducted at least every 2 weeks following therapy until cessation of enteral feeding.

Adequacy of Enteral Intake (AEI) was recorded at each review using the scale: AEI 0 = 0–24%, AEI 1 = 25–49%, AEI 2 = 50–74% and AEI 3 = 75–100% of daily nutritional needs, that is the contribution of enteral feeding to daily nutritional requirement. All patients were followed until their AEI was <1.

Speech pathology services were offered to all patients with oropharyngeal dysphagia to minimise aspiration and malnutrition risk. Videofluoroscopy and fibreoptic endoscopic evaluation of swallowing were available for at‐risk patients. Swallowing rehabilitation was not available to this patient cohort.

### Outcome measures

1. Weight loss during RT* between:
  - HRi and HIRi patients (All patients and FT only patients)
  - HRi and LIRi patients (All patients and FT only patients)
  - HIRi and LIRi patients (All patients and FT only patients),
2. Number of days from the commencement of radiation therapy until the commencement of Adequacy of Enteral Nutrition (AEI1) (i.e., enteral feeding reliance for 25–49% of nutritional needs) and AEI3 (i.e., enteral feeding reliance for 75–100% of nutritional needs) FT use between:
  - HRi and HIRi patients
  - HRi and LIRi patients
  - HIRi and LIRi patients

*Weight Loss during RT = % weight change between RT commencement and recorded weight in final week of RT.

### Statistical analyses

Statistical analysis was carried out using GraphPad Prism v7.02 (GraphPad Software Inc, San Diego, CA, USA). Descriptive statistics were calculated for baseline demographic characteristics, disease stage, treatment characteristics and potential prognostic factors that were analysed in the generation of the risk stratification model (Table 1). Each of these variables was available at the time of multidisciplinary tumour board meeting prior to the radiotherapy to allow timely risk stratification.

**Table 1:** Description of prognostic factors in patients with high, high‐intermediate and low‐intermediate risk for prolonged feeding tube use (*n* = 101).

Prognostic factor	Subgroup	High risk (HRi) FT Use^2^		High‐intermediate risk (HIRi) FT Use^3^		Low‐intermediate risk (LIRi) FT Use^4^		
		Yes/Total	%	Yes/Total	%	Yes/Total	%	*P* value^2^
Cancer site	Oropharynx	20/28	71%	16/31	52%	34/42	81%	0.008^1^
	Pharynx (other) or oral cavity	6/28	22%	6/31	19%	7/42	17%	
	Larynx, supraglottis	2/28	7%	9/31	29%	1/42	2%	
HPV Positive^5^		7/19	37%	8/14	57%	23/26	88%	0.001^1^
T‐stage	X, 0	0/28	0%	0/31	0%	1/42	2%	<0.001^1^
	1	0/28	0%	0/31	0%	17/42	41%	
	2	0/28	0%	0/31	0%	24/42	57%	
	3	19/28	68%	21/31	68%	0/42	0%	
	4	9/28	32%	10/31	32%	0/42	0%	
*N* stage	0	0/28	0%	18/31	58%	0/42	0%	<0.001^1^
	1	3/28	11%	4/31	13%	12/42	28%	
	2	23/28	82%	9/31	29%	28/42	67%	
	3	2/28	7%	0/31	0%	2/42	5%	
Bilateral neck node disease		15/28	54%	7/31	23%	10/42	24%	0.014^1^
Retropharyngeal node disease		6/28	21%	0/31	0%	2/42	5%	0.008^1^
Level 1 node disease		7/28	25%	6/31	19%	4/42	10%	0.215
Level 2 node disease		28/28	100%	0/31	0%	42/42	100%	<0.001^1^
Level 3 node disease		13/28	46%	6/31	19%	10/42	24%	0.047^1^
Level 4 node disease		9/28	32%	1/31	3%	1/42	2%	<0.001^1^
Level 5 node disease		4/28	14%	0/31	0%	6/42	14%	0.086
Concurrent chemotherapy		24/28	86%	17/31	55%	34/42	81%	0.011^1^
Dysphagia or odynophagia (pre‐existing)		11/28	39%	12/31	39%	1/42	2%	<0.001^1^
Nutrition (PG‐SGA)	Malnourished	5/28	18%	9/31	29%	7/42	17%	0.395
Body Mass Index^6^	Underweight (<18.5)	5/22	23%	5/31	16%	1/38	3%	0.049^1^
Age on commencing RT	≤ 65 years	18/28	64%	16/31	52%	35/42	83%	0.014^1^
Sex	Male	21/28	75%	24/31	77%	33/42	79%	0.941
ECOG Performance Status	0	9/28	32%	7/31	23%	26/42	62%	<0.001^1^
	1	18/28	64%	18/31	58%	16/42	38%	
	2	1/28	4%	6/31	19%	0/42	0%	
Charlson Comorbidity Index	0	12/28	43%	14/31	46%	30/42	71%	0.151
	1	4/28	14%	6/31	19%	4/42	10%	
	2	7/28	25%	5/31	16%	6/42	14%	
	3, 4, 5	5/28	18%	6/31	19%	2/42	5%	
Tobacco smoking^7^	Never or minimal	9/28	32%	7/30	23%	24/40	60%	0.003^1^
	Past	6/28	21%	13/30	44%	11/40	28%	
	Current	13/28	47%	10/30	33%	5/40	12%	
Alcohol drinker^8^	Never or social	19/28	68%	20/29	69%	32/39	82%	0.683
	Past	3/28	11%	3/29	10%	2/39	5%	
	Current	6/28	21%	6/29	21%	5/39	13%	

Two‐sided *P* value from Fisher’s exact test for difference between two subgroups, Pearson’s chi‐square test for difference between three or more unordered subgroups or Cochran–Armitage test for trend across 3 or more ordered subgroups.

^1^ Statistical significant difference *P* < 0.05.

^2^ ‘High risk (HRi) Feeding Tube use’ are patients with both T‐Stage ≥ 3 and level 2 node disease, with risk of feeding tube use for at least 25% of nutritional requirements.

^3^ ‘High‐intermediate risk (HIRi) Feeding Tube use’ are patients with T‐Stage ≥ 3 without level 2 node disease, with risk of feeding tube use for at least 25% of nutritional requirements.

^4^ ‘Low‐intermediate risk (HIRi) Feeding Tube use’ are patients without T‐Stage ≥ 3 with level 2 node disease, with risk of feeding tube use for at least 25% of nutritional requirements.

^5^ Human papilloma virus (HPV) status is restricted to patients with a diagnosis of cancer of the oropharynx only. 1 missing HPV status in HRi group, two in HIRi group and eight in LIRi group.

^6^ Body mass index (BMI): six patients missing BMI in HRi group and four in LIRi group.

^7^ Tobacco smoking: one patient missing tobacco smoking status in HIRi and 2 in LIRi group.

^8^ Alcohol drinker: two patients missing alcohol drinking status HIRi and 3 LIRi group.

For categorical variables, the frequency distribution between patients with HRi, HIRi and LIRi of prolonged feeding tube use was evaluated using Fisher’s exact test, the Cochran–Armitage test for trend if there were three or more ordered subgroups (eg. ECOG performance status) or the Pearson chi‐square test for three or more unordered subgroups (eg. cancer site). All *P*‐values were 2‐sided with a 0.05 α level of significance. Patients with unknown values for a particular factor were omitted from any models containing that factor.

## Results

One hundred and one patients treated with radical intent IMRT were eligible for inclusion in this study. They were categorised into HRi (*n* = 28), HIRi (*n* = 31) and LIRi (*n* = 42) of prolonged FT use prognostic groups. One (3.6%), seven (22.6%) and six (14.3%) patients did not have a FT inserted/adequately utilise their FT in the HRi, HIRi and LIRi risk groups, respectively. The majority of patients across each prognostic group were ≤65 years of age, with significantly more under 65 years old in the LIRi cohort (HRi: 64%; HIRi: 52%; LIRi: 88%, *P* = 0.014). Males were represented at a ratio of approximately 3:1 in each group. The most common cancer site was oropharynx, with a significantly larger proportion in the LIRi cohort (HRi: 71%, HIRi: 52%, LIRi: 81%, *P* = 0.008). 84% (59/70) of patients with OPC had a known human papilloma virus (HPV) status, with those in the LIRi cohort more likely to have HPV‐associated disease (88%, *P* = 0.001). Patients with never/minimal smoking history (*P* = 0.003), good performance status (*P* < 0.001), healthy body mass index (BMI) (*P* = 0.050) and no pre‐existing dysphagia (*P* < 0.001) were significantly more frequent within the LIRi prognostic group when compared to the HRi and HIRi cohorts. All patient demographic and tumour characteristics are shown in their entirety in the ‘Total’ column in Table 1.

There was no significant difference in weight loss between HRi or HIRi patients in total (HRi = 4.8% vs. HIRi = 5.2%, *P* = 0.813) or when using a FT (HRi = 4.6% vs. HIRi = 5.3%, *P* = 0.641). However, when compared with both the HRi and HIRi prognostic groups, LIRi patients lost significantly more weight in total (HRi = 4.8% vs. LIRi = 8.2%, *P* = 0.002; HIRi = 5.2% vs. LIRi = 8.2%, *P* = 0.006) and when using a FT (HRi = 4.6% vs. LIRi = 8.8%, *P* < 0.001; HIRi = 5.3% vs. LIRi = 8.8%, *P* = 0.002) (Table 2). No significant differences in days to commencement of AEI Levels 1 and 3 FT use were observed between each of the prognostic groups (Table 3).

**Table 2:** Comparison of weight loss across high risk (HR), high‐intermediate risk (HIR) and low‐intermediate risk (LIR) patients.

	High risk (HRi) of FT use vs. high‐intermediate risk (HIRi) of FT use^2^			High risk (HRi) of FT use vs. low‐intermediate risk (LIRi) of FT use^3^			High‐intermediate risk (HIRi) of FT use vs. low‐intermediate risk (LIRi) of FT use^4^		
	HRi (*n* = 28)	HIRi (*n* = 31)	*P*‐value	HRi (*n* = 28)	LIRi (*n* = 42)	*P*‐value	HIRi (*n* = 31)	LIRi (*n* = 42)	*P*‐value
% Weight Loss (All)	4.8 ± 4.8	5.2 ± 5.4	0.813	4.8 ± 4.8	8.2 ± 3.8	0.002^1^	5.2 ± 5.4	8.2 ± 3.8	0.006^1^

	HRi (*n* = 27)	HIRi (*n* = 24)		HRi (*n* = 27)	LIRi (*n* = 36)		HIRi (*n* = 24)	LIRi (*n* = 36)	
% Weight Loss (with FT)	4.6 ± 4.8	5.3 ± 5.0	0.641	4.6 ± 4.8	8.8 ± 3.6	<0.001^1^	5.3 ± 5.0	8.8 ± 3.6	0.002^1^

^1^ Statistical significant difference *P* < 0.05.

^2^ % Weight loss (i.e. % weight change between commencing radiotherapy and recorded weight in final week of radiotherapy) comparing patients at high risk (HRi) and high‐intermediate risk (HIRi) of feeding tube (FT) use (all patients and FT inserted only patients).

^3^ % Weight loss comparing patients at high risk (HRi) and low‐intermediate risk (LIRi) of feeding tube (FT) use (all patients and FT inserted only patients).

^4^ % Weight loss comparing patients at high‐intermediate risk (HIRi) and low‐intermediate risk (LIRi) of feeding tube (FT) use (all patients and FT inserted only patients).

**Table 3:** Days (mean) from the commencement of radiation therapy until the commencement of feeding tube (FT) use.^14^

	High risk (HRi) of FT use vs. high‐intermediate risk (HIRi) of FT use^1^			High risk (HRi) of FT use vs. low‐intermediate risk (LIRi) of FT use^2^			High‐intermediate risk (HIRi) of FT use vs. tow‐intermediate risk (LIRi) of FT use^3^		
	HRi (*n* = 27)	HIRi (*n* = 24)	*P*‐value	HRi (*n* = 27)	LIRi (*n* = 36)	*P*‐value	HIRi (*n* = 24)	LIRi (*n* = 36)	*P*‐value
Days to AEI1 (±SD)	23.4 ± 10.9	21.3 ± 15.2	0.568	23.4 ± 10.9	26 ± 11.8	0.378	21.3 ± 15.2	26 ± 11.8	0.183
Days to AEI3 (± SD)	30 ± 14.6	31.5 ± 33.9	0.840	30 ± 14.6	36.6 ± 13.3	0.075	31.5 ± 33.9	36.6 ± 13.3	0.441

^1^ ‘Feeding tube (FT) use’ means feeding tube was used for at least 25% of nutritional requirements (AEI1) and 75% of nutritional requirements (AEI3).

^2^ Days from commencement of radiation therapy until the commencement of feeding tube (FT) use (AEI1 and AEI3) – high risk (HRi) vs. high‐intermediate risk (HIRi) of feeding tube (FT) use.

^3^ Days from commencement of radiation therapy until the commencement of feeding tube (FT) use (AEI1 and AEI3) – high risk (HRi) vs. high‐intermediate risk (HIRi) of feeding tube (FT) use.

^4^ Days from commencement of radiation therapy until the commencement of feeding tube (FT) use (AEI1 and AEI3) – high risk (HRi) vs. high‐intermediate risk (HIRi) of feeding tube (FT) use.

## Discussion

Our previous body of work introduced a clinically useful risk stratification tool for both the requirement for and duration of significant FT use. The tool stratifies pharynx, oral cavity and supraglottic patients by two easily attainable clinical variables – T‐classification (<3 vs. ≥3) and presence/absence of involved level 2 lymph nodes – into four distinct risk classifications for the likelihood/intensity of FT use.^9^ This information is readily available when a patient is first presented at a multidisciplinary tumour board, with the model described capable of guiding decisions regarding prophylactic insertion of FTs. It does not take radiation dose factors into consideration.

Apart from cancer site, Anderson et al found advanced T‐classification to be the most significant prognostic factor for duration of FT use.^9^ This is not a new finding and is consistent with the observations of numerous published studies.^9^, ^12^, ^15^, ^16^, ^17^, ^18^ However, the contribution of level 2 lymphadenopathy to prolonged feeding tube use is a novel finding. The possible causality of level 2 nodal lymphadenopathy is detailed at length in this manuscript.^9^

Treatment‐induced weight loss and dehydration can lead to episodes of hospitalisation and treatment breaks, which adversely affect disease outcomes.^4^, ^19^, ^20^ Weight loss and deviations from planned body habitus have the potential to cause deviations in planned radiotherapy, that is less dose to the tumour and increased dose to healthy tissue. Greater sophistication in radiotherapy planning and delivery means less room for error, such as patient contour change as a result of weight loss. With the increasing conformality and subsequent precision of modern treatment techniques, such deviations from planned treatment geometry have potential for greater consequence to planned doses of radiation.^21^ Current practice dictates that such scenarios are often dealt with via adaptive radiotherapy protocols (i.e. radiotherapy planning is repeated to account for patient anatomical change), yet this is often not clinically feasible in busy, clinical departments where resources are stretched and modern technologies not always readily available.^22^, ^23^ Prevention of weight loss not only assists in patient well‐being, but also reduces the potential need for resource‐intensive adaptive radiation therapy. Optimal identification of at‐risk patients via a simple to use prognostic tool provides an opportunity to minimise the need for weight loss‐driven adaptation via instigation of timely, robust nutritional interventions.

The LIRi group were identified as patients with small primary tumours with level 2 nodal disease, so intuitively, presented with more OPC cases. Furthermore, an overwhelming majority presented with HPV‐associated disease (88%). Despite having a FT inserted, this patient cohort lost significantly more weight than those at higher risk of FT dependence. These findings are consistent with recent published data, who describe increased weight loss in HPV‐associated OPC patients at a similar magnitude to our work.^24^ Patients were also significantly younger, of good performance status, with a healthy BMI and no history of pre‐existing co‐morbidities such as underlying dysphagia. These patients reported to have never had or had a limited history of tobacco use. Of the 85% of patients with a known HPV status across the entire cohort, the presence of HPV‐associated disease was significantly higher in the LIRi group (LIRi: 88%; HRi: 37%; HIR: 57%).

Therefore, despite being a valuable resource in stratifying FT use, the findings of this weight loss analysis may necessitate the need for additional consideration (beyond the FT risk stratification tool) when a LIRi patient is identified. The risk stratification tool, alone, may be insufficient to fully characterise the FT requirements of this select patient cohort. This group loses more weight across their course of radiotherapy than those with a far more extensive disease burden. Sub‐optimal patient compliance to recommended FT use could provide an explanation for such weight loss.

More often than not, LIRi patients have a prophylactic FT inserted. Despite this, there remains some obvious, unmet needs with respect, but not limited, to dietetic counselling, optimal FT utilisation and psychological factors in the HPV‐associated head and neck cancer population, hindering FT utilisation and compromising optimal weight management. Similar studies suggest unmet needs, indicating the need for further investigation of underlying contributing factors.^25^, ^26^ This is further supported by the insignificant finding of days from the start of radiotherapy to the commencement of both AEI1 and AEI3 FT use – suggesting that LIRi patients are either using their FT, albeit inadequately, or providing an inaccurate account of their use upon weekly dietetic review. Additionally, a possible underlying clinician and allied health assumption of a well‐educated patient group capable of appropriate self‐management may further exacerbate the consequence of this non‐compliance. Conversely, despite the perception of increased self‐management capabilities, HPV‐associated cancer patients have higher levels of psychosocial and informational needs. If such needs become unmet, there is the potential to further complicate treatment and recovery.^27^, ^28^ All of these possible contributing factors must be the subject of further research, so that we, as the multidisciplinary team members responsible for the care of HPV‐associated OPC patients, can better understand their needs and attitudes towards their treatment and subsequent compliance to recommended nutritional advice. A push for future prospective studies is also supported by Vangelov et al.^24^ Further investigations may, perhaps, recommend nutritional support and guidance to the same level we apply to those patients we deem at highest risk of radiation‐induced dysphagia.

Oropharyngeal carcinoma has had a major demographic shift over the past two decades. The evolution of HPV‐associated OPC has introduced a paradigm shift in the traditionally atypical head and neck cancer patient (i.e. a patient that presents with a history of heavy alcohol and/or tobacco abuse).^29^, ^30^ Many western countries have witnessed a rise in the number of HPV‐associated cancers, compared with a previous population that included patients with predominantly carcinogen (tobacco and alcohol)‐associated disease.^31^ The United States reported a population‐level incidence increase of 225% in HPV‐positive OPC from 0.8 per 100,000 in 1988 to 2.6 per 100,000 in 2004. Alternatively, the incidence of HPV‐negative OPC decreased by 50% over the same period, from 2.0 per 100,000 to 1.0 per 100,000. This was further supported with a shift towards younger, white individuals.^30^, ^32^ HPV‐associated OPC has played a critical role in this demographic shift in disease incidence.^33^ The LIRi cohort identified in this study is representative of this growing number of patients with OPC presenting to radiotherapy departments. This particular cohort will continue to grow as HPV‐associated OPC numbers peak in the coming years.

A striking clinical feature of the HPV‐associated OPC patient is their excellent prognosis, with their risk of death halved in comparison with HPV‐negative patients.^34^, ^35^, ^36^ The concept of treatment de‐escalation is currently being reviewed at length, in order to minimise the risk of chronic treatment‐related toxicities in a patient cohort that, in general, has a favourable prognosis.^33^ Multiple treatment de‐intensification strategies are being investigated in each of the surgical, chemotherapy and radiotherapy (and combinations, thereof) disciplines. Reduced doses of radiation (to as low as 54Gy) are being investigated, minimising the risk of FT dependence that is often seen in patients receiving high doses of radiation to critical swallowing structures (i.e. pharyngeal constrictor muscles) that are in close proximity to macroscopic disease.^37^ Often, such regimens are coupled with less toxic cetuximab chemotherapy, compared with traditional cisplatin‐based regimes.^3^

The concept of radiation dose de‐escalation is relatively well established and accepted globally in low‐risk HPV‐associated OPC. Recent studies have reported equivalent outcomes to standard dose regimes.^38^ High‐tech radiotherapy has the capability for phenomenal dose sculpting, creating rapid dose fall off between target/tumour volumes and critical normal structures. Cautiously, we must therefore recognise that error apportioned to small uncertainties in radiotherapy treatment planning and delivery is higher than ever before. On‐treatment weight loss is one of these uncertainties that has the potential to alter the planned dose of radiotherapy via a change in patient geometry.^21^ In an era of radiation dose de‐escalation, it is incredibly important that the reduced dose being delivered is being delivered with precision. We are at a very real risk of further ‘de‐escalating’ dose that has already been ‘de‐escalated’ through variations apportioned to weight loss. Our study demonstrates that with weight loss at a heightened risk in the patient cohort most likely to be afforded such dose de‐escalation (i.e. HPV‐associated OPC or LIRi patients), we must recognise the extra supportive care measures required to ensure optimal weight maintenance is afforded this unique patient group.

In this cohort, no patient had access to swallowing rehabilitation. Furthermore, every effort was made to minimise patient pain. All patients were reviewed at least weekly by a medical doctor to prescribe analgesia in a stepwise fashion: mouthwashes and anti‐thrush measures, simple analgesia (e.g. soluble paracetamol), local anaesthetic mouthwashes (e.g. xylocaine and cocaine) and ultimately titration of opioids.^9^

This study has limitations inherent to a single‐institution, retrospective analysis. The authors recognise that the cohort of HPV‐associated patients is relatively small (38/70 patients with OPC), due to the availability of emerging technology enabling HPV diagnosis at the time this cohort received radiotherapy. Therefore, despite the identification of key clinical variables of HPV‐associated disease within the LIRi cohort, any conclusions must be interpreted with caution. We are unable to provide data on patients’ functional swallowing ability; however, we are able to accurately report on patients having oral, or partial oral, diet at various time points due to comprehensive, prospectively recorded nutritional data. All patients were treated by a single radiation oncologist; however, it must be acknowledged that these patients were treated over 8 years, a sufficient time period for even individual practice to vary. All patients were treated in an era with equitable access to FDG‐PET and IMRT, without swallowing exercises. This lends to uniformity in staging, volume delineation and treatment delivery across the cohort.

## Conclusion

Patients typically identified as low‐intermediate risk (LIRi) of prolonged FT use for ≥25% of nutritional requirement (T‐classification <3, level 2 node lymphadenopathy) report significantly increased weight loss compared with patients at higher risk of prolonged FT use undergoing definitive head and neck radiotherapy. This patient cohort demonstrates the demographic and diagnostic parameters of a stereotypical HPV‐associated OPC patient – characteristic of the changing landscape of the modern‐day head and neck radiotherapy patient. Results of this study suggest we should closely observe such patients throughout treatment, ensuring optimal weight maintenance and, in turn, facilitating precision radiotherapy. Larger, prospective studies are warranted to validate this finding and to examine any additional contributing factors – either physical or psychosocial – that may be contributing to the sub‐optimal weight management outcomes reported in this study.

## Conflict of interest

The author declares no conflict of interest.

## References

[1] De Luis DA , Izaola O , Aller R . Nutritional status in head and neck cancer patients. Eur Rev Med Pharmacol Sci 2007; 11: 239–43. 17876958

[2] Silver HJ , Dietrich MS , Murphy BA . Changes in body mass, energy balance, physical function, and inflammatory state in patients with locally advanced head and neck cancer treated with concurrent chemoradiation after low‐dose induction chemotherapy. Head Neck 2007; 29: 893–900. 17405169 10.1002/hed.20607

[3] van Wayenburg CA , Rasmussen‐Conrad EL , van den Berg MG , et al. Weight loss in head and neck cancer patients little noticed in general practice. J Prim Health Care 2010; 2: 16–21. 20690398

[4] Ohri N , Rapkin BD , Guha C , Kalnicki S , Garg M . Radiation therapy noncompliance and clinical outcomes in an urban academic cancer center. Int J Radiat Oncol Biol Phys 2016; 95: 563–70. 27020104 10.1016/j.ijrobp.2016.01.043

[5] Bourhis J , Sire C , Graff P , et al. Concomitant chemoradiotherapy versus acceleration of radiotherapy with or without concomitant chemotherapy in locally advanced head and neck carcinoma (GORTEC 99–02): An open‐label phase 3 randomised trial. Lancet Oncol 2012; 13: 145–53. 22261362 10.1016/S1470-2045(11)70346-1

[6] Driessen CM , Janssens GO , van der Graaf WT , et al. Toxicity and efficacy of accelerated radiotherapy with concurrent weekly cisplatin for locally advanced head and neck carcinoma. Head Neck 2015; 38: E559–E565. 25810154 10.1002/hed.24039

[7] Ho KF , Swindell R , Brammer CV . Dose intensity comparison between weekly and 3‐weekly Cisplatin delivered concurrently with radical radiotherapy for head and neck cancer: A retrospective comparison from New Cross Hospital, Wolverhampton, UK. Acta Oncol 2008; 47: 1513–18. 18607863 10.1080/02841860701846160

[8] Bossola M . Nutritional interventions in head and neck cancer patients undergoing chemoradiotherapy: A narrative review. Nutrients 2015; 7: 265–76. 25569622 10.3390/nu7010265 PMC4303838

[9] Anderson NJ , Jackson JE , Smith JG , et al. Pretreatment risk stratification of feeding tube use in patients treated with intensity‐modulated radiotherapy for head and neck cancer. Head Neck 2018; 40: 2181–192. 29756389 10.1002/hed.25316

[10] Christianen ME , Schilstra C , Beetz I , et al. Predictive modelling for swallowing dysfunction after primary (chemo)radiation: Results of a prospective observational study. Radiother Oncol 2012; 105: 107–14. 21907437 10.1016/j.radonc.2011.08.009

[11] Dirix P , Abbeel S , Vanstraelen B , Hermans R , Nuyts S . Dysphagia after chemoradiotherapy for head‐and‐neck squamous cell carcinoma: Dose‐effect relationships for the swallowing structures. Int J Radiat Oncol Biol Phys 2009; 75: 385–92. 19553033 10.1016/j.ijrobp.2008.11.041

[12] Bhayani MK , Hutcheson KA , Barringer DA , et al. Gastrostomy tube placement in patients with oropharyngeal carcinoma treated with radiotherapy or chemoradiotherapy: factors affecting placement and dependence. Head Neck 2013; 35: 1634–40. 23322563 10.1002/hed.23200

[13] Mallick I , Gupta SK , Ray R , et al. Predictors of weight loss during conformal radiotherapy for head and neck cancers – how important are planning target volumes? Clin Oncol (R Coll Radiol) 2013; 25: 557–63. 23651866 10.1016/j.clon.2013.04.003

[14] Poulsen MG , Riddle B , Keller J , Porceddu SV , Tripcony L . Predictors of acute grade 4 swallowing toxicity in patients with stages III and IV squamous carcinoma of the head and neck treated with radiotherapy alone. Radiother Oncol 2008; 87: 253–59. 18410976 10.1016/j.radonc.2008.03.010

[15] Cheng SS , Terrell JE , Bradford CR , et al. Variables associated with feeding tube placement in head and neck cancer. Arch Otolaryngol Head Neck Surg 2006; 132: 655–61. 16785412 10.1001/archotol.132.6.655

[16] Frowen J , Cotton S , Corry J , Perry A . Impact of demographics, tumor characteristics, and treatment factors on swallowing after (chemo)radiotherapy for head and neck cancer. Head Neck 2010; 32: 513–28. 19691115 10.1002/hed.21218

[17] Machtay M , Moughan J , Trotti A , et al. Factors associated with severe late toxicity after concurrent chemoradiation for locally advanced head and neck cancer: An RTOG analysis. J Clin Oncol 2008; 26: 3582–89. 18559875 10.1200/JCO.2007.14.8841 PMC4911537

[18] Wopken K , Bijl HP , van der Schaaf A , et al. Development of a multivariable normal tissue complication probability (NTCP) model for tube feeding dependence after curative radiotherapy/chemo‐radiotherapy in head and neck cancer. Radiother Oncol 2014; 113: 95–101. 25443500 10.1016/j.radonc.2014.09.013

[19] Capuano G , Grosso A , Gentile PC , et al. Influence of weight loss on outcomes in patients with head and neck cancer undergoing concomitant chemoradiotherapy. Head Neck 2008; 30: 503–8. 18098310 10.1002/hed.20737

[20] Russo G , Haddad R , Posner M , Machtay M . Radiation treatment breaks and ulcerative mucositis in head and neck cancer. Oncologist 2008; 13: 886–98. 18701763 10.1634/theoncologist.2008-0024

[21] Mali SB . Adaptive radiotherapy for head neck cancer. J Maxillofac Oral Surg 2016; 15: 549–54. 27833352 10.1007/s12663-016-0881-y PMC5083691

[22] Dawson LA , Sharpe MB . Image‐guided radiotherapy: Rationale, benefits, and limitations. Lancet Oncol 2006; 7: 848–58. 17012047 10.1016/S1470-2045(06)70904-4

[23] Brouwer CL , Steenbakkers RJ , Langendijk JA , Sijtsema NM . Identifying patients who may benefit from adaptive radiotherapy: Does the literature on anatomic and dosimetric changes in head and neck organs at risk during radiotherapy provide information to help? Radiother Oncol 2015; 115: 285–94. 26094076 10.1016/j.radonc.2015.05.018

[24] Vangelov B , Kotevski DP , Williams JR , Smee RI . The impact of HPV status on weight loss and feeding tube use in oropharyngeal carcinoma. Oral Oncol 2018; 79: 33–9. 29598948 10.1016/j.oraloncology.2018.02.012

[25] Fang CY . Heckman CJ . Informational and support needs of patients with head and neck cancer: current status and emerging issues. Cancers Head and Neck 2016; 1: 2–9. 10.1186/s41199-016-0017-6 PMC5488795 28670482

[26] Reich M , Leemans CR , Vermorken JB , et al. Best practices in the management of the psycho‐oncologic aspects of head and neck cancer patients: Recommendations from the European Head and Neck Cancer Society Make Sense Campaign. Ann Oncol 2014; 25: 2115–24. 24608199 10.1093/annonc/mdu105

[27] Gold D . The psychosocial care needs of patients with HPV‐related head and neck cancer. Otolaryngol Clin North Am 2012; 45: 879–97. 22793858 10.1016/j.otc.2012.05.001

[28] Hendry M , Pasterfield D , Gollins S , et al. Talking about human papillomavirus and cancer: Development of consultation guides through lay and professional stakeholder coproduction using qualitative, quantitative and secondary data. BMJ Open 2017; 7: e015413. 10.1136/bmjopen-2016-015413 PMC5734366 28652291

[29] Pytynia KB , Dahlstrom KR , Sturgis EM . Epidemiology of HPV‐associated oropharyngeal cancer. Oral Oncol 2014; 50: 380–6. 24461628 10.1016/j.oraloncology.2013.12.019 PMC4444216

[30] Chaturvedi AK , Engels EA , Pfeiffer RM , et al. Human papillomavirus and rising oropharyngeal cancer incidence in the United States. J Clin Oncol 2011; 29: 4294–301. 21969503 10.1200/JCO.2011.36.4596 PMC3221528

[31] Marur S , D'Souza G , Westra WH , Forastiere AA . HPV‐associated head and neck cancer: A virus‐related cancer epidemic. Lancet Oncol 2010; 11: 781–9. 20451455 10.1016/S1470-2045(10)70017-6 PMC5242182

[32] Zumsteg ZS , Cook‐Wiens G , Yoshida E , et al. Incidence of oropharyngeal cancer among elderly patients in the United States. JAMA Oncol 2016; 2: 1617–23. 27415639 10.1001/jamaoncol.2016.1804

[33] D'Souza G , Cullen K , Bowie J , Thorpe R , Fakhry C . Differences in oral sexual behaviors by gender, age, and race explain observed differences in prevalence of oral human papillomavirus infection. PLoS ONE 2014; 9: e86023. 24475067 10.1371/journal.pone.0086023 PMC3901667

[34] Mirghani H , Blanchard P . Treatment de‐escalation for HPV‐driven oropharyngeal cancer: Where do we stand? Clin Transl Radiat Oncol 2018; 8: 4–11. 29594236 10.1016/j.ctro.2017.10.005 PMC5862680

[35] Ang KK , Harris J , Wheeler R , et al. Human papillomavirus and survival of patients with oropharyngeal cancer. N Engl J Med 2010; 363: 24–35. 20530316 10.1056/NEJMoa0912217 PMC2943767

[36] Fakhry C , Westra WH , Li S , et al. Improved survival of patients with human papillomavirus‐positive head and neck squamous cell carcinoma in a prospective clinical trial. J Natl Cancer Inst 2008; 100: 261–9. 18270337 10.1093/jnci/djn011

[37] Sturgis EM , Ang KK . The epidemic of HPV‐associated oropharyngeal cancer is here: Is it time to change our treatment paradigms? J Natl Compr Canc Netw 2011; 9: 665–73. 21636538 10.6004/jnccn.2011.0055

[38] Gabani P , Lin AJ , Barnes J , et al. Radiation therapy dose de‐escalation compared to standard dose radiation therapy in definitive treatment of HPV‐positive oropharyngeal squamous cell carcinoma. Radiother Oncol 2019; 134: 81–8. 31005228 10.1016/j.radonc.2019.01.016

